# Editorial: Neural dynamics for brain-inspired control and computing: advances and applications

**DOI:** 10.3389/fnins.2025.1666218

**Published:** 2025-08-14

**Authors:** Mei Liu

**Affiliations:** Multi-scale Medical Robotics Center Limited, Hong Kong, Hong Kong SAR, China

**Keywords:** brain-inspired control, brain-like computing, human-machine interaction, brain-machine interfaces, neural dynamics, neural networks

The exploration of neural dynamics, inspired by the intricacies of brain functioning, has opened up new frontiers in the domains of control systems and computing. In recent years, neural dynamics has emerged as a pivotal framework for addressing complex, time-varying problems, offering both theoretical insights and practical solutions in brain-inspired control and computing systems. This Research Topic encapsulates the advances in neural dynamics and its applications, shedding light on innovative solutions to longstanding challenges in human-machine interaction, neuroscience data analysis, motor control, and brain-machine interface technologies.

At its core, neural dynamics draws upon the study of neural networks through dynamic systems, offering a promising avenue for decoding and modeling brain activities. Through brain-inspired models, we are now able to mimic and apply the dynamic principles governing cognitive and motor functions, thereby opening new doors to computational neuroscience, intelligent control systems, and interactive technologies. The ability of neural dynamics to process and decode neural signals with resilience to noise has placed it at the forefront of emerging technologies, such as brain-machine interfaces, which are central to future advancements in neurotechnology and rehabilitation.

This Research Topic features four carefully selected contributions that highlight the current advancements, as well as the challenges that lie ahead in the field. These articles delve into the theoretical underpinnings of neural dynamics and its practical applications in brain-inspired control and computing:

1. **Exploring the suitability of piecewise-linear dynamical system models for cognitive neural dynamics**: this article investigates the potential of piecewise-linear dynamical systems to model brain dynamics, particularly in cognitive tasks. The study compares piecewise-linear models with traditional linear models, emphasizing their potential for real-time brain-state control and neuromodulation applications (Wu et al.).
2. **AM-MTEEG: multi-task EEG classification based on impulsive associative memory**: addressing the challenge of cross-subject variability in EEG-based brain-computer interfaces (BCIs), this research introduces AM-MTEEG, a multi-task model that integrates convolutional networks and impulsive associative memory to improve EEG classification accuracy and reduce subject-specific performance variance (Li et al.).
3. **Application of deconvolutional networks for feature interpretability in epilepsy detection**: this contribution presents a novel convolutional neural network (CNN) model for epilepsy detection, integrating Squeeze-and-Excitation modules to enhance interpretability. The model successfully quantifies the contributions of individual EEG channels, offering valuable insights into seizure detection (Shao et al.).
4. **A review of epilepsy detection and prediction methods based on EEG signal processing and deep learning**: this comprehensive review surveys the current landscape of deep learning-based approaches for epilepsy detection, categorizing various signal processing methods and network designs. It offers key insights into improving prediction cycles and emphasizes the importance of patient-independent datasets for more robust models (Zhang et al.).

Despite the significant strides made in neural dynamics for brain-inspired applications, several challenges remain. Model simplification, stability, and computational demands are still critical issues, particularly when dealing with high-dimensional data and noisy neural signals. Moreover, the integration of these advanced models into practical applications, such as brain-machine interfaces, requires overcoming hurdles in real-time processing and interpretation.

Theoretical advancements, particularly in understanding brain cognition and motor functions, continue to fuel the development of more sophisticated neural dynamics models. Our collective understanding of brain-inspired control algorithms, including cerebellar-like motor control and neural networks for human-machine interaction, is steadily improving. However, ongoing research is essential to enhance model stability, interpretability, and efficiency.

As we continue to explore and refine the applications of neural dynamics, we are presented with the exciting potential to develop more intuitive and effective brain-machine interfaces, facilitating advancements in areas such as cognitive rehabilitation and real-time neurofeedback. The research featured in this collection represents a significant step forward, but much work remains to ensure that these technologies reach their full potential.

We encourage further exploration of these principles, as the field of brain-inspired control and computing holds great promise for shaping the future of human-machine collaboration and brain-like intelligence.

